# Electrolyte Effects on Disorder-Enhanced Capacitance in Nanoporous Carbons

**DOI:** 10.1021/acselectrochem.5c00472

**Published:** 2026-01-20

**Authors:** Xinyu Liu, Kara Fong, Zhaohan Shen, Wei Yu, Hirotomo Nishihara, Clare P. Grey, Alexander C. Forse

**Affiliations:** † Yusuf Hamied Department of Chemistry, 2152 University of Cambridge, Cambridge CB2 1EW, U.K.; ‡ Institute of Multidisciplinary Research for Advanced Materials, 13101 Tohoku University, Sendai 980-8577, Japan; § Advanced Institute for Materials Research (WPI-AIMR), Tohoku University, Sendai 980-8577, Japan; ∥ Frontier Research Institute for Interdisciplinary Sciences (FRIS), Tohoku University, Sendai 980-8578, Japan

**Keywords:** nanoporous carbon, electrochemical double layer capacitor, structural disorder, ion adsorption capacity, quantum capacitance

## Abstract

The impact of pore structure and surface functionality on the capacitance of nanoporous carbons has been widely studied across different electrolytes, yet the role of electrolyte chemistry in structural disorder-driven and ion adsorption capacity-related capacitance remains largely unexplored. In this study, we investigate the relationship between capacitance and the degree of structural order in 20 nanoporous carbons using ionic liquid electrolytes, aiming to establish the generality of disorder-driven capacitance and explore its underlying mechanisms. Our results demonstrate that carbons with smaller graphene-like domains and larger ion adsorption capacities exhibit higher capacitance in 1-ethyl-3-methylimidazolium tetrafluoroborate (EMIBF_4_) ionic liquid, consistent with our previous findings in 1 M tetraethylammonium tetrafluoroborate (TEABF_4_) in acetonitrile (ACN). More generally, we find that the capacitance of a given carbon remains similar across different ionic liquid and organic electrolytes, provided that the pores are accessible to the electrolyte ions. This study shows the generality of disorder-driven and adsorption-dependent capacitance in nanoporous carbons in organic and ionic liquid systems and suggests that factors such as the nature of the defects and how they affect quantum capacitance may play an important role in disorder-driven capacitance, ultimately providing insights for designing high-performance supercapacitor electrodes.

## Graphical abstract

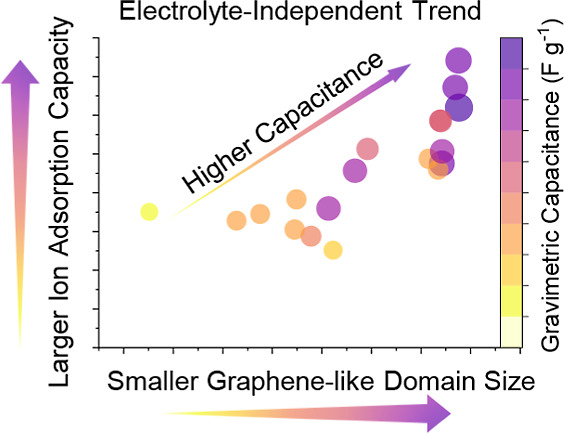

## Introduction

Electrochemical double layer capacitors (EDLCs) are promising energy storage devices with fast charging–discharging capability and long cycle lives, bridging the gap between traditional capacitors and batteries.^1,2^ Nanoporous carbons, especially activated carbons, are the cheapest and most widely used electrode materials in commercial EDLCs.^3^ These materials consist of defective graphene-like domains which form the pore walls of three-dimensional porous structures with a distribution of pore sizes, predominantly below 2 nm for microporous carbons.^4–7^ Previous studies have extensively investigated the effects of pore structures (including surface area and average pore size)^5,8–13^ and surface functionality^14–17^ on the capacitive performance of microporous carbons across different electrolytes. However, clear design principles for making nanoporous carbons with superior performance are still lacking due to their complex structures.

Recently, our study of 20 nanoporous carbons showed that the capacitance in the organic electrolyte tetraethylammonium tetrafluoroborate (TEABF_4_) (1 M in acetonitrile) is correlated with the domain sizes of the graphene-like sheets within the carbon electrodes, as probed by solid state nuclear magnetic resonance (NMR) spectroscopy experiments and simulations.^18^ In this approach, the local structural order degree is measured by the Δδ value derived from NMR experiments on electrolyte-saturated carbon samples, Δδ being defined as the chemical shift difference between “in-pore” and neat electrolyte resonances

1 $$\Delta \delta \;\left(ppm\right)={\delta_{in-pore}-\delta }_{neatelectrolyte}$$

where δ_neatelectrolyte_ is the chemical shift of the free electrolyte and δ_in‑pore_ is the chemical shift of “in-pore” resonance.^15,19–24^ For predominantly microporous carbons, the magnitude of Δδ provides a measure of the size of the ordered domain^19^ and representative ordered domain areas can be extracted by a previously reported simulation approach.^25^ It was found that nanoporous carbons with smaller graphene-like domain sizes (i.e. with a more disordered local structure) have higher capacitance.^18^ With an additional series of synthesised carbons, our recent study revealed that both ordered domain sizes and ion adsorption capacities significantly influence capacitance.^26^ Specifically, nanoporous carbons with smaller graphene-like ordered domains and high ion adsorption capacities demonstrate enhanced capacitance.

Our findings were supported by Raman spectroscopy.^27^ Raman spectra of disordered nanoporous carbons contain two major peaks: the D band (between 1330 and 1350 cm^–1^), attributed to the A_g_^1^ breathing mode of the six membered carbon rings in a graphene sheet, which becomes allowed (observed) when there is structural disorder, and the *G* band (between 1580 and 1590 cm^–1^), arising from the *E*_g_^2^ stretching mode of the sp^2^ bonds.^28,29^ The intensity (peak height) ratio of these peaks (I_D_/I_G_) is a measure of the carbon disorder, decreasing with smaller graphene-like domain sizes, as described by the 3-stage model proposed by Robertson and Ferrari.^28^ Our work demonstrated that nanoporous carbons with higher capacitance have smaller I_D_/I_G_ values and D-band full width half maxima (FWHM),^27^ consistent with our observations based on NMR spectroscopy experiments and simulations, suggesting that carbons with smaller graphene-like domains have higher capacitances, as measured in our experiments in symmetric EDLCs in a conventional organic electrolyte 1 M tetraethylammonium tetrafluoroborate in acetonitrile (1 M TEABF_4_/ACN).

Previous studies have extensively investigated the impact of electrolyte ion size on capacitance performance in supercapacitors, showing that the capacitance can be enhanced by optimising ion accessibility to micropores.^30–32^ However, it is unclear whether the disorder-driven capacitance arises from specific interactions between smaller graphene-like domains and certain cations, anions, or solvent molecules—i.e., whether this effect is electrolyte-specific. To address this, we investigate the role of the electrolyte in disorder-driven capacitance across organic electrolytes and ionic liquids with different anions and cations. Our results show that carbons with smaller graphene-like domain sizes and higher ion adsorption capacities generally have higher capacitance in ionic liquids and organic electrolytes with varying cation–anion combinations, provided that the ions can access the carbon nanopores. Three-electrode measurements show that no specific interaction was found between the carbons and particular cations or anions. Instead, the capacitance is found to be more dependent on the structure of the carbons, rather than on the electrolyte composition. Our work establishes the generality of disorder-enhanced capacitance in both organic electrolytes and ionic liquid regardless of the presence of solvent and highlights ion adsorption capacity as a key descriptor alongside structural disorder. Together, these findings propose a clear design pathway for nanoporous carbons with improved capacitance across different electrolytes.

## Experimental Section

### Materials

Commercial activated carbons (YP-50F, YP-80F from Kuraray; PW-400, SC-1800, ACS-PC from Carbon Activated Corp.; EL-104, EL-106 from Jacobi) and activated carbon cloths (ACC-10, ACC-15, ACC-20 from Kynol) were used as received. Thermally annealed carbons were prepared by heating the pristine carbons (ACS-PC and EL-104) under argon flow (60 cm^3^/min) at temperatures of 700–1200 °C for 5 h (heating rate: 5 °C/min). Carbon powders were made into self-standing films for use as electrodes, while carbon cloths were used directly. Ionic liquids (EMIBF_4_, EMITFSI, SEt_3_TFSI from IoLiTec) were dried at room temperature under dynamic vacuum for 1 week before use. See detailed experimental methods in Supporting Information.

### Electrochemical Measurements

Symmetric two-electrode CR2032 coin cells were assembled in a N_2_-filled glovebox. Electrodes (diameter: 0.64 cm) had identical masses within 0.2 mg (3–7 mg total) with mass loadings of 10.9–16.3 mg/cm^2^ for carbon films and 17.2–21.8 mg/cm^2^ for carbon cloths. A glass fiber separator (diameter: 1.43 cm) was placed between electrodes with around 150 μL electrolyte. Cyclic voltammetry was performed at 10 mV/s (0–2.5 V). Galvanostatic charge–discharge measurements were conducted at current densities of 0.05–1 A/g (0–2.5 V) using a Biologic BCS-805 potentiostat. Capacitance was calculated from the slope of the second half of the discharge curve. At least two cells were prepared for each carbon, with error bars representing standard deviations between repeat cells.

Three-electrode measurements were performed in Swagelok T-cells with working electrodes (diameter: 0.47 cm), oversized YP-80F counter electrodes (≥4 × working electrode mass), and Ag wire pseudo-reference electrodes in 750 μL electrolyte. Cells were pre-cycled for 20 cycles at 2 mV/s before measurements at 0.05 A/g (±1 V vs *E*_ocv_). See Supporting Information for more details.

### NMR Spectroscopy

Carbon films (∼5 mg) were dried at 100 °C under vacuum for more than 24 h, then soaked with EMIBF_4_ (around 150 μL) for at least 24 h to achieve equilibrium saturation before packing into 2.5 mm rotors. ^19^F MAS NMR spectra were acquired at 9.4 T with 5 kHz spinning using a 90° pulse-acquire sequence with recycle delays longer than 5 ×T_1_ (typically 8–12 s). Spectra were referenced to hexafluorobenzene at −164.9 ppm. See Supporting Information for deconvolution of NMR spectra.

### Additional Characterization

X-ray photoelectron spectroscopy (XPS) was performed using a Thermo Fisher K-Alpha spectrometer with Al-Kα source after degassing samples at <5 × 10^–7^ bar for 90 min. Atomic compositions were averaged from 2–3 spots per sample. Raman spectroscopy was conducted with a Renishaw inVia microscope (532 nm laser, 2.5 mW, 10 s acquisition). Powder X-ray diffraction (XRD) was performed using a Rigaku MiniFlex600 with Cu Kα radiation (40 kV, 40 mA, 5° min^–1^, 10°–90°). Temperature-programmed desorption (TPD) measurements were performed using a home-made system consisting of an induction heating unit and quadrupole mass spectrometer. Carbon samples (1–2 mg) were heated from ambient temperature to 1800 °C at 10 °C/min under high vacuum, and the evolved gases (H_2_, H_2_O, CO, CO_2_) were quantified using calibrated mass spectrometry to determine functional groups. More details are included in the Supporting Information.

## Results and Discussion

The capacitances of a series of commercial activated carbons were investigated in EMIBF_4_, with two of them (ACS-PC and EL-104) additionally studied after thermal annealing under argon at different temperatures (700–1200 °C), as described previously.^18^ These heat-treated carbons were labelled APC-X °Cs and AEL-Y °Cs, with “X °C” and “Y °C” representing the annealing temperature. “A” indicates that the carbon was annealed.

All the carbons show similar trends in an EMIBF_4_ ionic liquid to those in 1 M TEABF_4_ (ACN) at both low and high current densities (Figures 1a,b and S1–S3), suggesting that the identify of the carbon structure, rather than the electrolyte, is the dominant factor in determining capacitance. At the lower current of 0.05 A/g, among the commercial nanoporous carbons, ACS-PC and SC-1800 exhibit the highest capacitance, reaching 138 and 126 F/g, respectively, whereas PW-400 shows the lowest capacitance of 94 F/g. YP-50F, YP-80F, EL-104 and EL-106 demonstrate intermediate capacitance values of around 100 F/g. Upon thermal annealing, the capacitances decreased for both thermally annealed ACS-PC and EL-104 as the annealing temperature increased (from 138 F/g for pristine ACS-PC to 99 F/g for APC-1100 °C, an ACS-PC sample thermally annealed at 1100 °C, and from 99 F/g for pristine EL-104 to 88 F/g for AEL-1200 °C), aligning with previous findings in 1 M TEABF_4_ (ACN).^18^ It is worth noting, however, that the capacitance values are generally higher in EMIBF_4_ than in 1 M TEABF_4_ (ACN) for all the studied carbons at the lower current density (with the slope of *y* = k*x less than 1) (Figure 1a), potentially due to more efficient charge storage in EMIBF_4_ in the absence of a solvent, the ionic liquid eliminating solvation shells and enhancing ion packing within the carbon nanopores.^24,33^ At 1 A/g, the capacitance values in EMIBF_4_ are closer to those in 1 M TEABF_4_ (ACN) for the studied carbons, likely due to the higher viscosity and lower ionic conductivity of EMIBF_4_ (60 mS cm^–1^ for 1 M TEABF_4_/ACN and 14 mS cm^–1^ for EMIBF_4_ at room temperature),^34^ which limits ion transport and leads to a greater capacitance drop at high current densities (Figures 1b and S3). Overall, the capacitance values for all the studied carbons in EMIBF_4_ are similar to those in 1 M TEABF_4_ (ACN) at both low and high current densities, as indicated by the slope of correlation (*y* = k*x) being close to 1 (Figure 1a,b), although some rate-dependent differences are observed at higher current densities.

**Figure 1:**
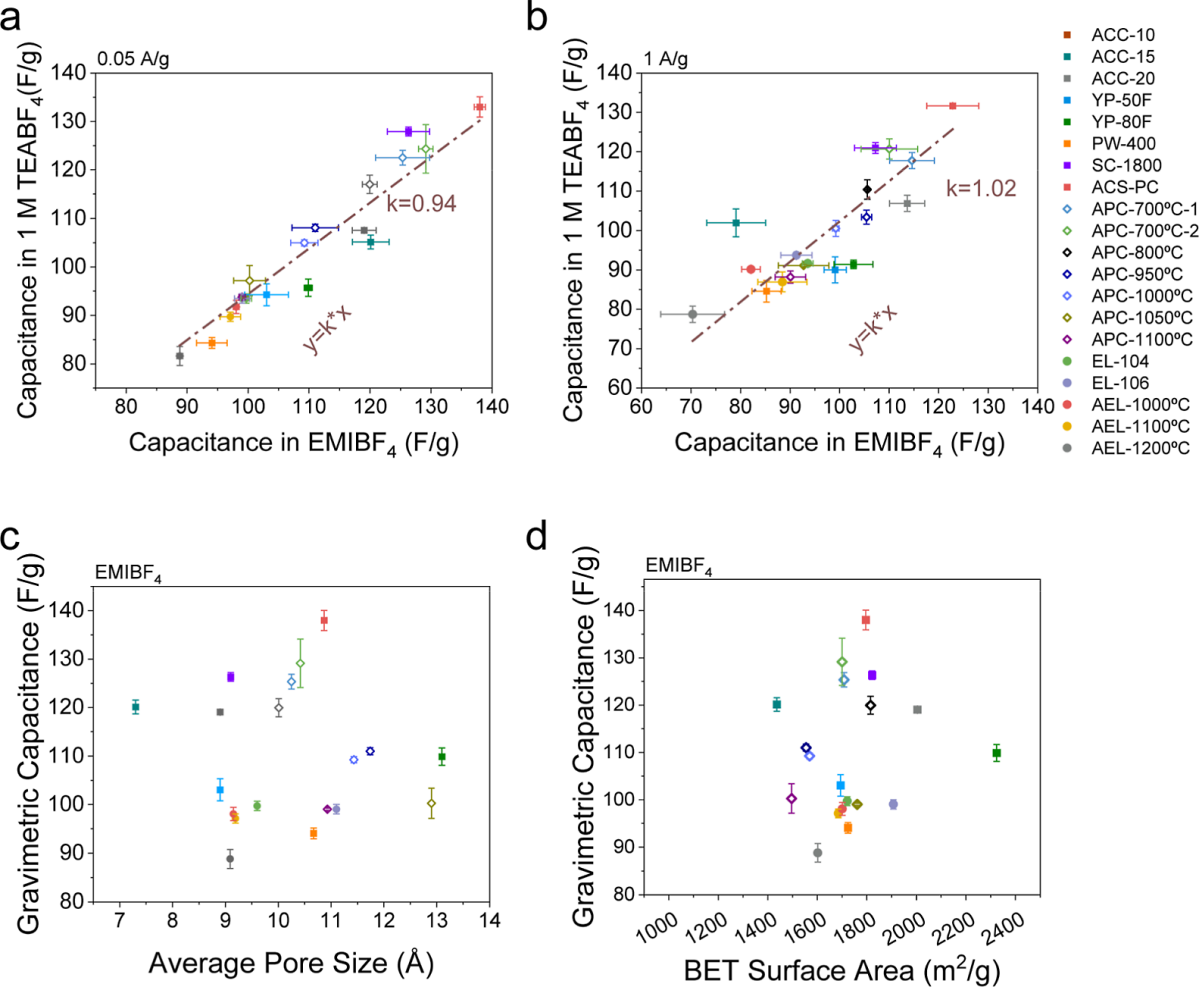
Relationships between the gravimetric capacitances for the studied carbons in the EMIBF_4_ ionic liquid and 1 M TEABF_4_ (ACN) measured (a) at 0.05 A/g and (b) at 1 A/g, in a two-electrode symmetric coin cell configuration. “APC-X °C” and “AEL-Y °C” represent thermally annealed ACS-PC and EL-104, respectively, with “X °C” and “Y °C” demonstrating the annealing temperature. (c) Relationship between gravimetric capacitance in EMIBF_4_ at 0.05 A/g and average pore size of the studied carbons. The average pore sizes were calculated from the pore size distributions, which were derived from the N_2_ physisorption isotherms based on quenched solid density functional theory (QSDFT).^35^ (d) Relationship between gravimetric capacitance in EMIBF_4_ at 0.05 A/g and BET surface area of the studied carbons. The results of BET surface area, average pore size and capacitance values in 1 M TEABF_4_ (ACN) were taken from our previous work, Copyright [2024] The American Association for the Advancement of Science.^18^ All electrochemical measurements were conducted at room temperature.

In addition, five of the commercial activated carbons were tested in another ionic liquid electrolyte, 1-ethyl-3-methylimidazolium bis­(trifluoromethylsulfonyl)­imide (EMITFSI), which has a different anion (Figure S4). The overall trend remains similar across all three electrolytes. It is clear that for a given carbon, the capacitance remains similar in the organic electrolyte and ionic liquids. Minor variations in absolute capacitance values suggest that while electrolyte properties (e.g., dielectric constant, size of the ions) may introduce subtle effects, the overall capacitance at low currents is primarily governed by the carbon rather than the choice of electrolyte.

To further investigate the relationship between capacitance and porosity in an electrolyte with well-defined ion sizes (EMIBF_4_ without solvent), the capacitance in EMIBF_4_ of all the studied nanoporous carbons at 0.05 A/g was plotted against the average pore size (Figure 1c) and BET surface area (Figure 1d and Table S1). Although pore size can play a role in ionic liquid systems where strong ion pairing affects the effective size of charge-carrying species,^36,37^ no correlation is observed between capacitance and pore size at either low (Figure 1c,d) or high (Figure S5) current densities, consistent with our previous findings in 1 M TEABF_4_ (ACN)^18^ and prior studies in ionic liquids.^13^ Furthermore, even among carbons with nearly identical pore size distributions (Figure S1E), capacitances vary significantly from 94 to 138 F/g in EMIBF_4_, demonstrating that pore size is not the dominant factor determining capacitance in our studied carbons. Additionally, no correlation is shown between the capacitance and oxygen content measured from X-ray photoelectron spectroscopy (XPS) (Figure S6A), while a weak positive correlation is observed with oxygen content measured by temperature-programmed desorption (TPD) experiments, though with considerable scatter (Figure S6B). It is worth noting that XPS assesses surface and sub-surface oxygen content while TPD reflects the oxygen-containing groups in the bulk material.^38,39^ This suggests that similar to the behaviour observed in 1 M TEABF_4_ (ACN), other structural factors beyond the pore sizes from gas sorption and surface functionality govern the capacitance for the studied nanoporous carbons. We note that the presence of a high concentration of oxygen-containing functional groups may often be associated with residual disorder in the structure.

Magic angle spinning (MAS) NMR spectra of EMIBF_4_-saturated carbon samples (with sufficient soaking time to achieve equilibrium saturation^24,40^ ) show at least two resonances (Figure 2a) similar to our previous studies.^15,18,20^ The higher frequency (left-hand) resonances with chemical shifts close to the neat electrolyte are attributed to “ex-pore” anions and the lower frequency (right-hand) peaks are assigned to the “in-pore” anions.^15,18,21,22,24^ The variations in in-pore peak line widths reflect differences in ion exchange rates^41^ and distributions of local chemical environments within the pore structures.^33^ Deconvolution of NMR spectra revealed a correlation between the gravimetric capacitance and ^19^F Δδ values in EMIBF_4_ for the commercial activated carbons (Figures 2b and S7, Table S2). Nanoporous carbons with smaller magnitudes of Δδ values generally show higher capacitance in EMIBF_4_, consistent with our previous observations in 1 M TEABF_4_ (ACN).^18^ Similarly, the thermally annealed ACS-PC and EL-104 (APC-X °Cs and AEL-Y °Cs) exhibit decreasing capacitances as annealing temperature increased, with Δδ values increasing in magnitude (from approximately −1.85 ppm for pristine ACS-PC to −2.77 ppm for the same carbon annealed at 1100 °C (APC-1100 °C); and from around −6.8 ppm for EL-104 to −11.2 ppm for AEL-1200 °C) (Figure 2c). However, the increase in Δδ values for APC carbons upon annealing is less significant in EMIBF_4_, with a difference of less than 1 ppm between ACS-PC and APC-1100 °C, compared to 1.3 ppm difference in 1 M TEABF_4_ (ACN) (Figure 2d). This could be attributed to the slower exchange effects between in-pore and ex-pore species due to the higher viscosity of EMIBF_4_ or differences in average distances between the BF_4_^−^ ions and the carbon sheets between the two electrolytes, leading to slightly different Δδ values. It is worth noting that the Δδ value is predominantly affected by the structure of the carbon (sizes of graphene-like domains for predominantly microporous carbons),^19^ rather than the electrolyte ions or probe molecules used. Raman spectroscopy measurements further show that carbons with smaller *I*_D_/*I*_G_ peak height ratios (Figures 2e and S8) and larger D-band full width half maxima (FWHM) (Figure S8) have higher capacitance, suggesting that carbons with smaller graphene-like domains have larger capacitances when the EMIBF_4_ electrolyte is used, aligning with our previous findings in 1 M TEABF_4_ (ACN).^18,27^ Note that while Raman measures the structural disorder, it does not provide information on whether the ions can access the pores (unlike NMR, see below). For example, a highly disordered carbon may have a low capacitance if there is no interconnected pore structure.

**Figure 2:**
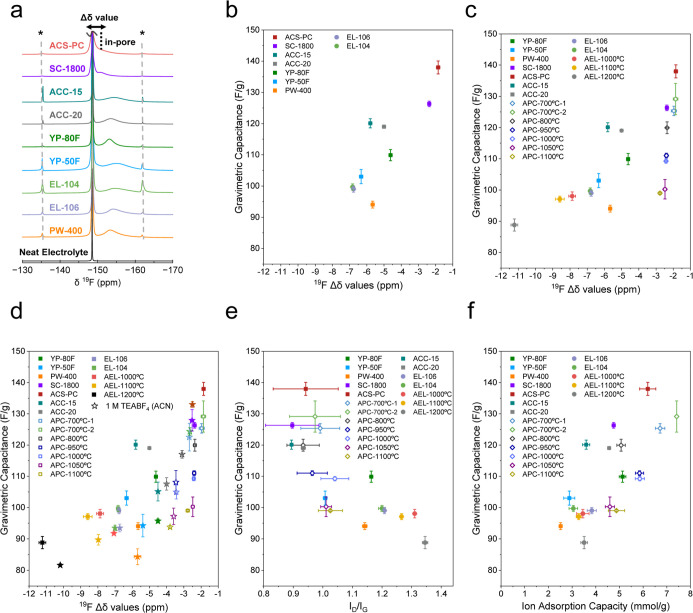
(a) ^19^F MAS NMR spectra (9.4 T, 5 kHz MAS) of the commercial nanoporous carbons soaked with EMIBF_4_. “*” represents the spinning side bands. Relationship between gravimetric capacitance (measured at 0.05 A/g in two electrode symmetric coin cell configuration) and ^19^F Δδ values of (b) the commercial nanoporous carbons in EMIBF_4_ derived from (a), with the in-pore chemical shifts taken as the weighted average for carbons showing multiple in-pore environments, all the studied nanoporous carbons including the thermally annealed carbons (c). See Figure S7 for a correlation between volumetric capacitance and ^19^F Δδ values. (d) Relationship between gravimetric capacitance and ^19^F Δδ values of all studied carbons in EMIBF_4_ (squares), with previous data in 1 M TEABF_4_ (ACN) (stars) added for comparison, with the same colour coding of the carbons for the two electrolytes. Copyright [2024] The American Association for the Advancement of Science^18^ (e) Relationship between gravimetric capacitance in EMIBF_4_ and *I*_D_/*I*_G_ values from Raman measurements for the studied nanoporous carbons. (f) Relationship between gravimetric capacitance and ion adsorption capacities in EMIBF_4_ from NMR measurements for the studied nanoporous carbons. The results of capacitance, ^19^F Δδ values in 1 M TEABF_4_ (ACN) and *I*_D_/*I*_G_ values are from our previous work.^18,27^

In addition to the graphene-like domain sizes, the NMR measurements simultaneously probe the ion adsorption capacity of the carbons in the absence of an applied potential, based on the relative proportion of ex-pore to in-pore resonances. A correlation is observed between gravimetric capacitance and ion adsorption capacity in EMIBF_4_ for commercial nanoporous carbons and their thermally annealed counterparts (Figures 2f and S9). The best-performing carbons ACS-PC, APC-700 °C, and APC-700 °C_2 have the highest ion adsorption capacities of around 7 mmol/g in EMIBF_4_, whereas PW-400, the sample with the lowest capacitance, shows a significantly lower ion adsorption capacity of around 2.5 mmol/g (Figure 2f). The ion adsorption capacities in EMIBF_4_ are also substantially higher than those observed in 1 M TEABF_4_ (ACN) (around 0.8–1 mmol/g) for all the studied carbons (Figure S10). Interestingly, this correlation between capacitance and ion adsorption capacity is less evident in 1 M TEABF_4_ (ACN) (Figure S10), likely due to solvation effects in organic electrolytes. In organic electrolytes with solvent present, carbons can adsorb various amounts of solvent prior to charging, and some solvent may leave the pores as the applied potential is varied.^5^ As a result, the total ion adsorption capacity remains low in the absence of charge, leading to weaker correlation between capacitance and ion adsorption capacity. In contrast, for ionic liquids, where solvent effects are absent, only the electrolyte ions exist in the system, and they can either enter the pores or not. Therefore, nanoporous carbons with larger ion adsorption capacity generally have higher capacitance in EMIBF_4_, as the charge is stored based on the accumulation of ions within the nanopores. Overall, these findings (Figure 2) suggest that both the ion adsorption capacity of the ionic liquid and graphene-like domain sizes are key factors determining capacitance in nanoporous carbons in ionic liquid electrolyte. Notably, when the studied carbons show similar ion adsorption capacities in 1 M TEABF_4_ (ACN), the graphene-like domain size becomes the dominant factor, and vice versa (Figures S10 and 2d). Ion adsorption capacity measured by NMR does not correlate with N_2_-derived pore volume (Figure S10C), suggesting that the accessibility of pores to large electrolyte ions differs fundamentally from N_2_ molecule adsorption at 77 K.

To further explore the interaction between nanoporous carbons and electrolyte ions, three-electrode measurements were conducted on two selected commercial activated carbons with different degrees of structural order, i.e. ACS-PC (more disordered) and EL-104 (more ordered) in various electrolytes. Both carbons exhibit pure double-layer capacitive behaviour in all studied electrolytes (Figure S11). At 0.05 A/g, the gravimetric capacitances at positively and negatively charged electrodes are similar in 1 M TEABF_4_ (ACN) and 1 M LiTFSI (ACN) for both carbons (Figure 3a). Notably, despite the differences in electrolyte ions, the overall capacitance of each carbon electrode remained similar (Figure 3a). Furthermore, the capacitance difference between the two studied carbons in three-electrode measurements closely mirrors the value observed in two-electrode configurations (around 43 F/g in 1 M TEABF_4_ (ACN) at 0.05 A/g).

**Figure 3:**
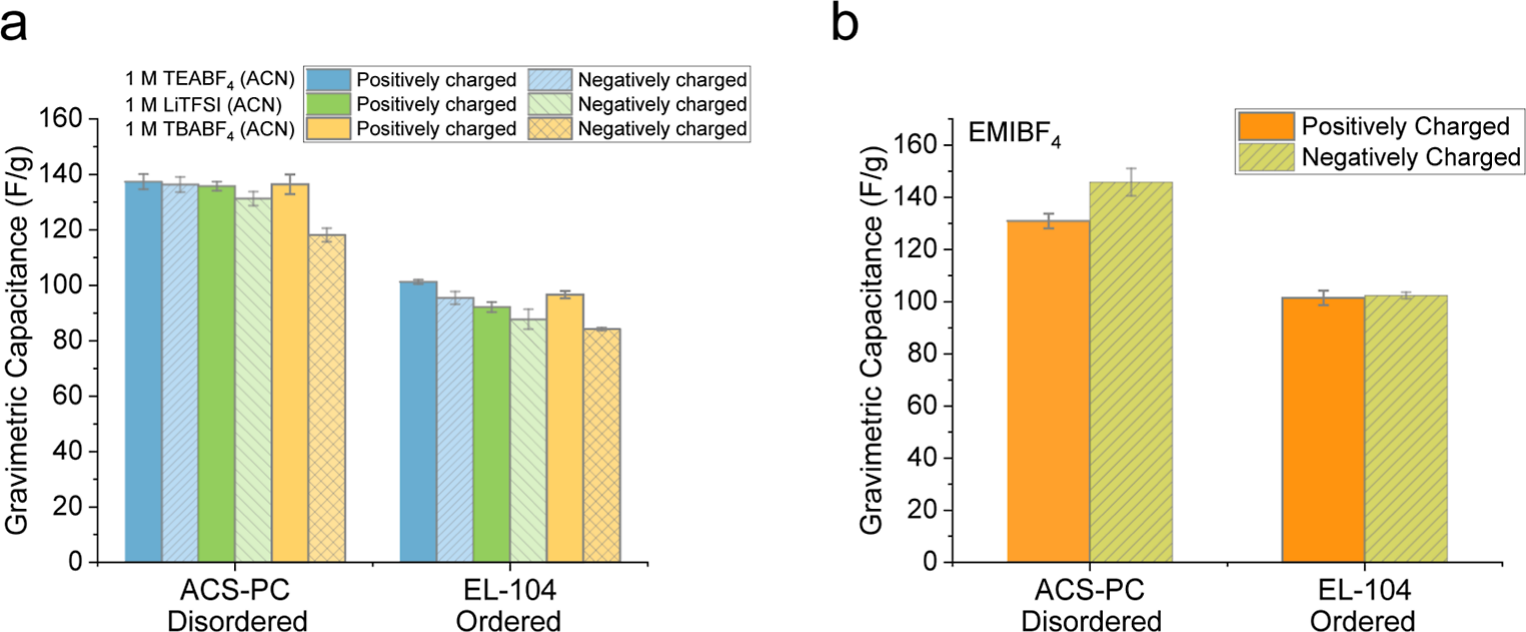
(a) Gravimetric capacitance of ACS-PC and EL-104 calculated from constant charge–discharge measurements at 0.05 A/g in a three-electrode cell in 1 M TEABF_4_ (ACN), 1 M LiTFSI (ACN) and 1 M TBABF_4_ (ACN). (b) Gravimetric capacitances of ACS-PC and EL-104 calculated from constant charge–discharge measurements at 0.05 A/g in a three-electrode cell in EMIBF_4_. See Table S3 for the ion sizes of different electrolytes.

In 1 M tetrabutylammonium tetrafluoroborate (TBABF_4_), the cation size is evidently larger (Table S3 TBA^+^: bare ion diameter: 0.82 nm; solvated: 1.44 nm)^42^ compared to other electrolyte ions and is comparable to the average pore size of the studied carbons (around 1 nm).^18^ Therefore, a slight decrease in capacitance is observed at the negatively charged electrodes for both carbons (purple bars in Figure 3a), indicating that the charge compensation mechanism partially relies on counterion adsorption for both carbons. On the other hand, at the positively charged electrodes, the capacitances in 1 M TBABF_4_ (ACN) remained similar to those in 1 M TEABF_4_ (ACN) and 1 M LiTFSI (ACN) for both carbons (Figure 3a), suggesting that the charge compensation mechanism is dominated by counterion adsorption for the positively charged electrodes. Based on these findings, the effect of structural disorder on capacitance is observed across all studied electrolytes, indicating no preferential behaviour towards specific cations or anions. Instead, the capacitance is primarily dominated by the intrinsic carbon structure, except in cases where the ion size impedes pore accessibility, as shown in the capacitance decrease at the negatively charged electrodes in 1 M TBABF_4_ (ACN) (Figure 3a) and also in previous studies.^30,31^

Similar results were observed in the ionic liquid EMIBF_4_ (Figure 3b). For a given carbon, the capacitance remained similar for both the positively charged and negatively charged electrodes, although the carbon with smaller graphene-like domains (ACS-PC) exhibits a slightly higher capacitance (∼15 F/g) at the negatively charged electrode compared to positively charged electrode in EMIBF_4_, whereas EL-104 showed similar capacitance values at both electrodes. This slight increase in the capacitance of the negatively charged electrode for ACS-PC is only observed in EMI^+^-containing ionic liquids without the presence of solvents (Figure S12). We hypothesize that the disordered carbon has slightly stronger interactions with EMI^+^ ions which can efficiently screen the electrode charge in the negative electrode, and this effect becomes evident only in the absence of organic solvent (Figure S12). In general, our results demonstrate that capacitance in both organic electrolytes and ionic liquids is primarily dependent on carbon structures than on specific electrolyte ions, provided that the ions can freely access the carbon nanopores.

To rationalise these results, we consider a model in which the total capacitance (*C*_total_) arises from two terms which are generally separated as follows: the capacitance associated with an electrical double layer (*C*_EDL_) and the quantum capacitance of the electrode (*C*_Q_) associated with filling discrete (quantised) energy levels with electrons or holes.^43^ These capacitances combine in series according to

2 $$\frac{1}{C_{total}}=\frac{1}{C_{EDL}}+\frac{1}{C_{Q}}$$

where we note that the smaller of the two capacitances dominates the total capacitance. The quantum capacitance reflects the electronic structure of the electrode and increases with the electrode’s density of states near the Fermi level, *N*(*E*_F_).^44^ For metallic electrodes, where there is a continuous and high density of states near *E*_F_, the quantum capacitance is large, and thus its contribution to *C*_total_ is negligible, and the capacitance is governed by the electrical double layer term (i.e. the nature of the ion packing/desolvation with the pores or at the surfaces of the electrode). With carbon-based electrodes, however, *C*_Q_ may become comparable to or much smaller than that of *C*_EDL_. In devices based on single-layer graphene, for example, both theoretical^44^ and experimental^17,45–47^ studies have suggested that the low values of *C*_Q_ limit the total capacitance across a range of aqueous electrolytes and ionic liquids, with the quantum and therefore total capacitances dropping to close to zero at 0 V (at the Dirac point). Given that the capacitance measured herein is largely independent of the electrolyte and driven primarily by the carbon structure, we hypothesize that the change in quantum capacitance between our different carbon electrodes may strongly influence the total capacitance. Indeed, this hypothesis may also explain why capacitance increases with the degree of carbon disorder. Although there is some disagreement about the role that disorder plays on electronic structure, many previous studies have demonstrated that disorder in carbon-based materials—whether arising from defects,^48–50^ doping,^49,51^ strain,^52^ or local curvature/pore structure^52,53^ —increases the density of states near *E*_F_ and, consequently, the quantum capacitance, particularly at low voltages.

Powder X-ray diffraction measurements show that all studied carbons exhibit broad (002) peaks (Figure S13), indicating highly disordered structures with limited turbostratic stacking, with a distribution of spacings between the disordered graphene layers rather than extended graphitic order. Experimental studies on model carbons demonstrate that such limited stacking has only modest effects on quantum capacitance and does not compensate for the reduction in gravimetric capacitance caused by the increased material density and reduced surface area associated with stacking.^54^ It is, therefore, unlikely that the more graphitic regions with more extended stacking plays a more important role in controlling the capacitance seen here. In the carbons studied here, quantum capacitance may overlap or be synonymous with pseudocapacitive behaviour caused by the redox activity of defects caused by the presence of functional groups, the filling of defect-associated electronic states being charge-compensated by ions in the double layer/pores, albeit with different charge screening characteristics. Overall, we propose that the limited quantum capacitance of more ordered carbon electrodes gives rise to their lower capacitances, while the larger quantum capacitance of more-disordered carbons accounts for their enhanced capacitances.

## Conclusions

In conclusion, our work demonstrates that both structural disorder and ion adsorption capacities are key descriptors of capacitance across various electrolytes, extending our previous findings in 1 M TEABF_4_ (ACN) to ionic liquid EMIBF_4_ and other organic electrolytes. With a combination of NMR and Raman spectroscopy, we found that the capacitance in EMIBF_4_ strongly correlates with ^19^F Δδ values and *I*_D_/*I*_G_ ratios for 20 nanoporous carbon samples, suggesting that carbons with smaller graphene-like domains have higher capacitance in both electrolytes. In addition, a correlation was observed between capacitance in EMIBF_4_ and ion adsorption capacity without the applied potential. More generally the structural disorder-enhanced capacitance is consistently observed in other organic electrolytes and ionic liquids with different cations–anion combinations, as long as the electrolyte ions can access the carbon nanopores. No specific interactions between carbon disorder and particular cations or anions were observed in three-electrode measurements, reinforcing that capacitance is primarily governed by the intrinsic structure of the carbons, rather than the electrolyte composition. These findings suggest that it is important to consider the role of defects in increasing the density of states and furthermore, that the role of quantum capacitance in controlling disorder-driven capacitance should be explored further. Overall this work provides clear design principles for optimising carbon-based supercapacitor electrodes across various electrolytes.

## Supplementary Material



## Data Availability

All data are available in the main text or the Supporting Information materials. All raw experimental data files are available in the Cambridge Research Repository, Apollo. DOI: 10.17863/CAM.122968

## Notes

Additional experimental details, materials, and methods, electrochemical data (CV, GCD, capacitance in different electrolytes), relationship between gravimetric capacitance and pore size, functional group, and Raman-derived parameters, relationship between capacitance and ion adsorption capacity in different electrolytes, table of pore sizes, pore volume, BET surface area, ^19^F Δδ value, and electrolyte ion sizes (PDF)

## #

Division of Chemistry and Chemical Engineering, California Institute of Technology, Pasadena, California 91125, United States
