# Supplementary material for: Electrolyte Effects on Disorder-Enhanced Capacitance in Nanoporous Carbons
Source: ACS Electrochem. 2026 Jan 20;2(2):510–8. doi: 10.1021/acselectrochem.5c00472 (PMC12884468; doi:10.1021/acselectrochem.5c00472)
Supplement: Supplementary file 1 [file ec5c00472_si_001.pdf]

Supporting Information: **Electrolyte Effects on Disorder-enhanced Capacitance in Nanoporous Carbons**

Xinyu Liu,[1] Kara Fong,[1] Zhaohan Shen[2], Wei Yu[3,4], Hirotomo Nishihara[2,3], Clare P. Grey,[1*] Alexander C. Forse[1*]

1 Yusuf Hamied Department of Chemistry, University of Cambridge, Cambridge CB2 1EW, U.K.

2. Institute of Multidisciplinary Research for Advanced Materials, Tohoku University, Sendai, 980-8577, Japan.

3. Advanced Institute for Materials Research (WPI-AIMR), Tohoku University, Sendai, 980-8577, Japan

4. Frontier Research Institute for Interdisciplinary Sciences (FRIS), Tohoku University, Sendai 980-8578, Japan.

* Corresponding Authors: Clare P. Grey (cpg27@cam.ac.uk) and Alexander C. Forse (acf50@cam.ac.uk)

Contents

Methods....3

Supplementary Figures....7

Supplementary Tables....22

Supplementary References....25

Methods

Materials

All the commercial nanoporous carbons and their thermally annealed counterparts were the same as the ones used in our previous work.[1] YP50F and YP80F activated carbon powders were obtained from Kuraray (Japan), PW-400 activated carbon, SC-1800 activated carbon and ACS porous carbon (ACS-PC) powders were obtained from Carbon Activated Corp. (America), Puragen Activated Carbon, Advanced Chemicals Supplier Material, respectively. Activated carbon cloths ACC-5092-10, ACC-5092-15 and ACC-5092-20 (ACC10, ACC15 and ACC20) were purchased from Kynol. PowerSorb EL-106 and PowerSorb EL-104 activated carbon powders were obtained from Jacobi (Jacobi Carbons Group).

The annealed carbons were treated with a Carbolite tube furnace in a horizontal ceramic tube under a set temperature with Ar gas flow at 60 cm^3/min. The ramp of temperature was 5 ℃/min until the set temperature was reached. The set temperature remained constant for 5 hrs and then samples were cooled down to room temperature under flowing argon. APC-700ºC-2 was annealed for 1 hr. The carbon powders were evenly distributed in a 15 ml SRX61 round bottom alumina boat crucible (Almath Crucibles Ltd) and placed in the centre of the furnace, where the temperature experienced by the carbons was the set temperature. The tube was purged with Ar gas flow for a minimum of 15 min before starting to increase the temperature. The carbon powders were made into self-standing films as electrodes. The carbon cloths were directly used as electrodes. The ionic liquids, 1-ethyl-3-methylimidazolium tetrafluoroborate ($EMIBF_4$), 1-ethyl-3-methylimidazolium bis(trifluoromethanesulfonyl)imide (EMITFSI) and triethylsulfonium bis(trifluoromethylsulfonyl)imide (SEt_3TFSI) were purchased from IoLiTec Ionic Liquids Technologies. $EMIBF_4$, EMITFSI and SEt_3TFSI were all dried at room temperature under a dynamic vacuum for a week before being taken into a N_2-filled glovebox.

Coin Cells and Electrochemical Measurements

All the coin cells were two-electrode cells (CR2032), and were assembled in a N_2-filled glovebox. The coin cells were symmetric (*i.e.,* both electrodes were the same carbon material). The electrodes were cut using a stainless-steel manual punching cutter (diameter 0.64 cm, Hilka Tools), and the two electrodes in each cell had identical masses within 0.2 mg. The masses of the electrodes ranged from around 3 mg to 7 mg). For activated carbon film electrodes, the mass loading of the electrodes varied from 10.9 to 16.3 mg/cm^2, with an approximate thickness of 0.25 mm. For activated carbon cloths, the mass loading varied from 17.2 to 21.8 mg/cm^2, with an approximate thickness of 0.5 mm.

Two electrodes were placed onto a coin cell bottom casing with a larger diameter (1.43 cm) glass fibre separator placed between the electrodes. Around 150 µL of the electrolyte was added around the separator with a Pasteur pipette. After the electrolyte fully wetted the electrodes, two SS316 spacer disks were placed directly on top of the electrode, serving as current collectors. Additionally, a single SS316 spring was placed above the spacing disks. The top casing was then used to close the coin cell, with all coin cells parts made of stainless steel. The coin cells were sealed with a Compact Hydraulic Coin Cell Crimper (Cambridge Energy Solutions) under a pressure of 80 kg/cm^3 for approximately 40 s. All coin cells parts were supplied by Cambridge Energy Solutions.

All electrochemical measurements were conducted in a two-electrode configuration with a Biologic BCS-805 potentiostat. Cyclic voltammograms of the cells were first obtained at a scan rate of 10 mV/s with a fixed potential window of 0-2.5 V for at least 5 cycles. For constant charge-discharge measurements, each cell was measured sequentially under different current densities at 0.05, 0.1, 0.2, 0.5, 0.75, and 1 A/g with a voltage window of 0-2.5 V for at least 3 cycles under each current density at room temperature. For each type of carbon, at least two cells were made. The capacitance of each cell was calculated through galvanostatic charge discharge measurements from the slope of the second half of the discharge curve in the last cycle. This method to calculate the capacitance of the single electrode assumes that the capacitance of two electrodes is equal. The error bars in corresponding figures represent the standard deviations between the repetitive cells. See our previous work for more details on calculations of the gravimetric and volumetric capacitance.[1]

Three-electrode Swagelok Cells

All electrochemical measurements were conducted in a three-electrode configuration with a potentiostat (VSP-3e and VMP-3e, Biologic). The electrochemical cells for three-electrode experiments were assembled in a 1/4'' Swagelok T-shaped-cell. The working electrodes were cut with a "3/16" inch puncher. The counter electrodes were YP-80F films cut with "1/4" inch puncher with mass at least four times larger than the working electrodes. The reference electrode is a "pseudo" reference electrode, which is a piece of flattened Ag wire (diameter of around 1 mm). The three-electrode Swagelok cell was assembled with an excess amount of electrolyte of 750 µL in a N_2-filled glovebox to ensure that the Ag wire was immersed in electrolyte solution. The whole cell was tightened manually with spanners. All three-electrode Swagelok cells were pre-cycled with cyclic voltammetry experiments for 20 cycles at a scan rate of 2 mV/s with a potential window of +0.2 V+E_{ocv} to -0.2 V+E_{ocv}.

Cyclic voltammograms of the cells were first obtained at a scan rate of 2 mV/s with a fixed potential window of 0-2 V (+1 V+E_{ocv} to -1 V+E_{ocv}) for at least 5 cycles. For constant charge-discharge measurements, each cell was measured under different current densities at 0.05 A/g with a voltage window of 0-2 V (+1 V+E_{ocv} to -1 V+E_{ocv}) for at least 3 cycles The capacitance of each cell was calculated through galvanostatic charge discharge measurements from the slope of the whole discharge curve in the last cycle.

Solid-state NMR Spectroscopy Experiments

Each carbon sample was made into a carbon film as described above and dried for at least 24 h at 100 °C in vacuo before being transferred to a N_2-filled glovebox. The film was then cut and weighed in the glovebox. The weighed film piece (around 5 mg) was put into a sealed vial overnight (at least 24 hours) with $EMIBF_4$ to fully saturate the sample (around 150 µL) before being packed into 2.5 mm rotors. This 24-hour equilibration period has been shown to be sufficient to achieve equilibrium saturation from previous studies.[2, 3]The vial was sealed with PTFE tape on the vial thread and a layer of electrical tape on the outside. The rotors were quickly packed (typically within 3 minutes). For each sample, the rotor was weighed before and after being packed to calculate the mass of the electrolyte added to the system. Excess electrolyte was carefully removed with tissue papers after packing.

NMR spectroscopy experiments were carried out with a Bruker Avance Neo spectrometer in a Bruker 2.5 mm HX double resonance probe. Measurements were carried out at a magnetic field strength of

9.4 T, corresponding to a ^{1}H Larmor frequency of 400.1 MHz. All spectra were acquired with a 90° pulse-acquire sequence at a sample spinning speed of 5 kHz. Recycle delays were set to be more than five times T_1 for each sample to ensure that the experiments were quantitative. The recycle delays are typically 8-12 s. ^{19}F NMR spectra were referenced relative to neat hexafluorobenzene (C_6F_6) at −164.9 ppm as a secondary reference. See our previous paper for details on deconvolution of the NMR spectra.[1]

X-ray Photoelectron Spectroscopy

X-ray photoelectron spectroscopy (XPS) was conducted under an ultrahigh vacuum condition (< 10^{-9} mbar) using a Thermo Fisher Scientific K-Alpha X-ray photoelectron spectrometer equipped with an Al-Kα monochromated X-ray source. All carbon samples were degassed under a high vacuum condition (< 5×10^{-7} bar) for 90 mins to remove adsorbed oxygen before the measurement, and then pasted onto the specific testing stage using a double-sided conductive carbon tape. Survey scans were measured with 200 eV pass energy, 1 eV step size and 200 ms (10 ms × 20 scans) dwell time. Atomic compositions were calculated and averaged according to the spectra acquired from 2-3 different spots on each sample, with error bars representing the standard deviation between these measurements. The XPS results of the studied nanoporous carbons were adapted from our previous study.[1]

Raman Spectroscopy

The Raman spectroscopy measurements results were adapted from our previous work. See our previous work for a detailed discussion of the Raman spectroscopy measurements.[4]

Raman spectroscopy was carried out with a Renishaw inVia confocal Raman microscope equipped with a 532 nm laser. The spectra were recorded with an acquisition time of 10 s and two accumulations using a laser output power of 2.5 mW. The maximum 532 nm laser power is 500 mW and the spectra were collected using 0.5 % power. The averaged Raman spectra were averaged over three to four spectra at different spots for each sample. The measurements were conducted by Jaehoon Choi and Dr. Simon Fleischmann in Helmholtz-Institute (HIU).

For four-peak analysis, all Raman spectra were normalised by maximum intensity, baseline-subtracted and deconvoluted using a four-peak model with Lorentzian function. Fit parameters included peak positions, full widths at half maximum (FWHM), and peak areas. Each spectrum was deconvoluted for three times by three different researchers from HIU and Cambridge. For each fitting, different initial peak positions were applied, then varied freely to achieve the optimal fitting. The results in following sections represent the average of the three fits, with the error bars showing the standard deviation among the different fits. For four-peak analysis with constraints, the position of the left hand "shoulder" peak was kept consistent between 1180 to 1200 cm^{-1}, and the position of the right hand "shoulder was maintained at 1500 to 1530 cm^{-1} for all spectra. The full widths at half maximum (FWHM) and peak areas were varied freely to achieve the optimal fitting.

Temperature Programmed Desorption (TPD)

The home-made Temperature-Programmed-Desorption (TPD) system consists of a reaction unit and a gas analysis unit, as described in previous work.[5] The reaction unit consists of a high-frequency

induction heating system (EASYHEAT 8310LI, Ambrell), a graphite holder inside a quartz chamber with circulating cooling water and a radiation thermometer to measure the holder temperature. A holder made of high-purity graphite (PYROGRAPH, Toyo Tanso, 99.9999%) was used. In this work, the temperature of the holder was measured from the bottom side through a quartz window using the new setup reported in our recent work to improve the sensitivity of temperature detection.[6] The gas analysis unit consists of a quadrupole mass spectrometer (QMS, MPH-100M, Inficon Co., Ltd.), a gas reservoir for calibration, and a stainless steel high-vacuum line pumped by a turbo molecular pump (TMP).

Before each TPD test, the empty sample holder was heat treated at 1800 ℃ under high vacuum for 1 hour to clean the surface and ensure that no quantifiable amounts of gas were released during the subsequent TPD test. The weight of the activated carbon sample (about 1-2 mg) for each TPD test was measured using a high-precision electronic balance (Sartorius SE2) and placed in the graphite sample holder. Then the reaction unit is vacuumed to 2×10^{-6} Pa and heated from ambient temperature to 1800 ℃ at 10 ℃ per minute. The gases (H_2, H_2O, CO and CO_2) released during heating are quantified using a calibrated QMS where calibration gases are supplied from a gas reservoir to establish a response curve correlating the intensity of the detector signal with known gas concentrations.

X-Ray Diffraction (XRD)

XRD of carbon powder was performed using a MiniFlex600 diffractometer (Rigaku) with Cu Kα radiation generated at 40 kV and 40 mA, at a scanning speed of 5.0 deg min^{-1} over a range of 10° to 90°.

Supplementary Figures

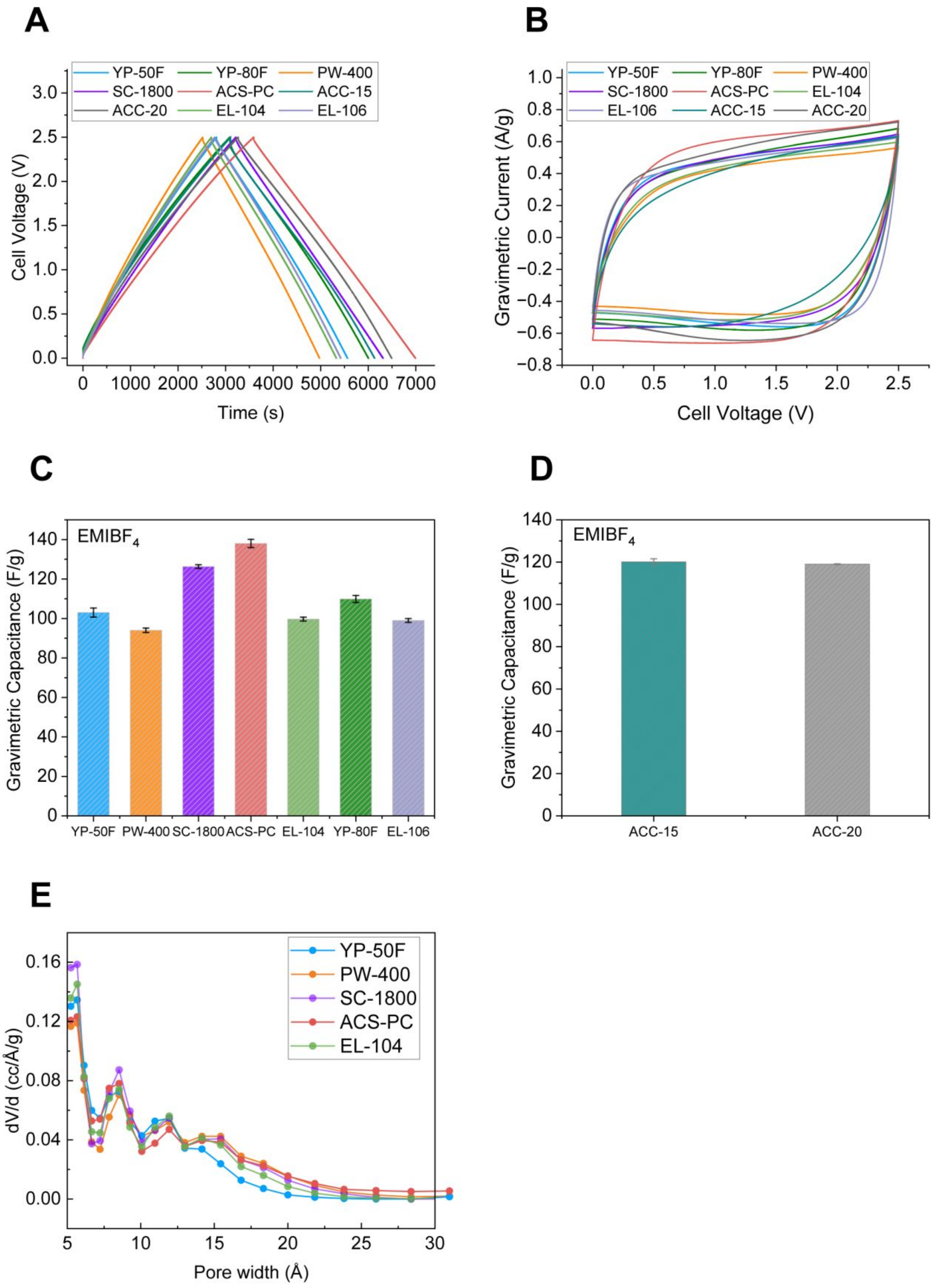

Figure S1: Galvanostatic charge−discharge plots, gravimetric capacitance of the studied commercial activated carbons and pore size distribution plot of commercial activated carbons. (**A**) Galvanostatic charge−discharge plots of 7 commercial activated carbons and 2

activated carbon cloths in $EMIBF_4$ at 0.05 A/g. **(B)** Cyclic voltammograms of 7 commercial activated carbons and 2 activated carbon cloths in $EMIBF_4$ at 10 mV/s. **(C)** Gravimetric capacitance of 7 commercial activated carbons in $EMIBF_4$ calculated from discharge curve from **(A)** at 0.05 A/g. **(D)** Gravimetric capacitance of 2 activated carbon cloths in $EMIBF_4$ calculated from discharge curve from **(A)** at 0.05 A/g. (**E**) Pore size distribution plots of five commercial activated carbons from N_2 gas physisorption isotherms derived from quenched solid functional theory density (QSDFT), based on slit-pore model. Data reproduced from a previous study.[1]

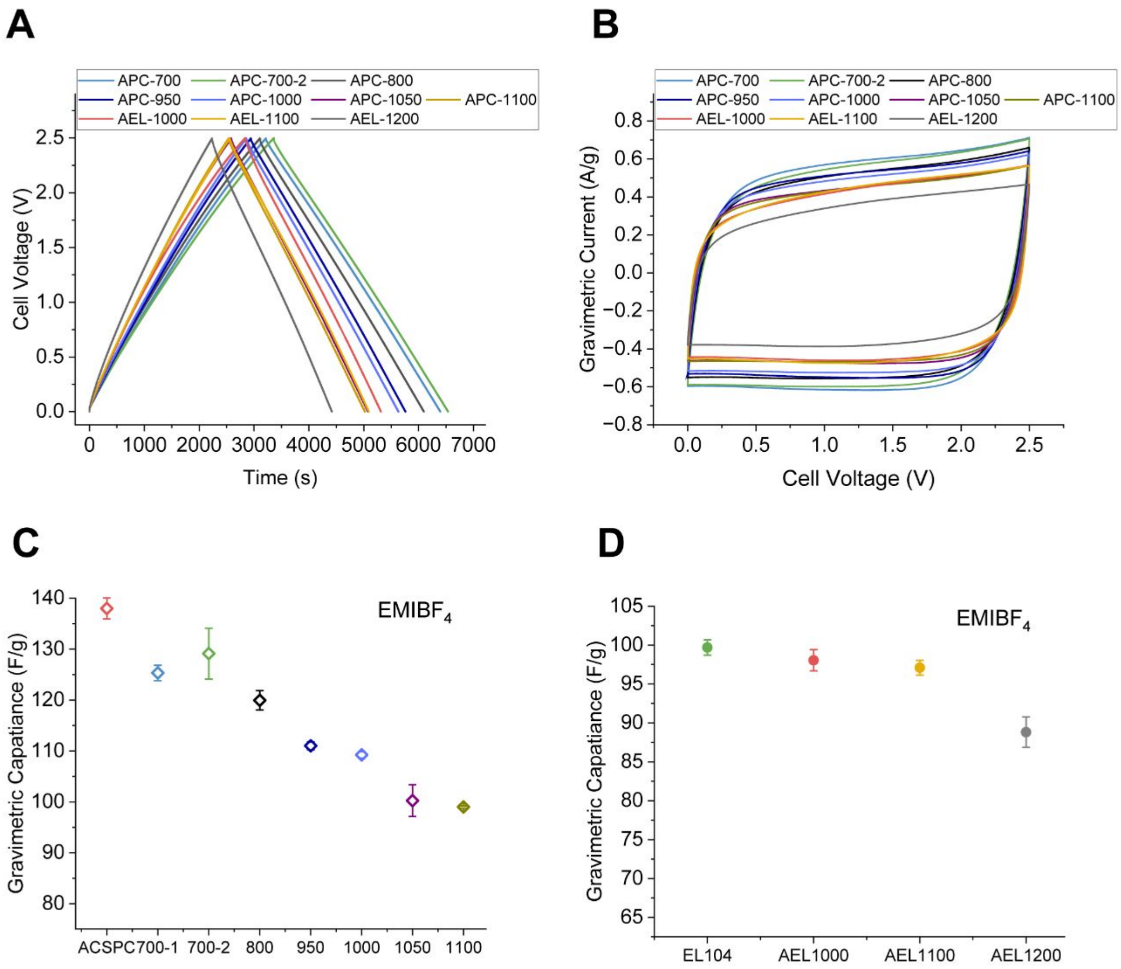

Figure S2: Galvanostatic charge−discharge plots and gravimetric capacitance of the thermally annealed carbons. (**A**) Galvanostatic charge−discharge plots of the thermally annealed carbons in $EMIBF_4$ at 0.05 A/g. **(B)** Cyclic voltammograms of the thermally annealed carbons in $EMIBF_4$ at 10 mV/s. **(C)** Gravimetric capacitance of pristine ACS-PC and thermally annealed ACS-PCs in $EMIBF_4$ calculated from discharge curve from **(A)** at 0.05 A/g. **(D)** Gravimetric capacitance of pristine EL-104 and thermally annealed EL-104s in $EMIBF_4$ calculated from discharge curve from **(A)** at 0.05 A/g.

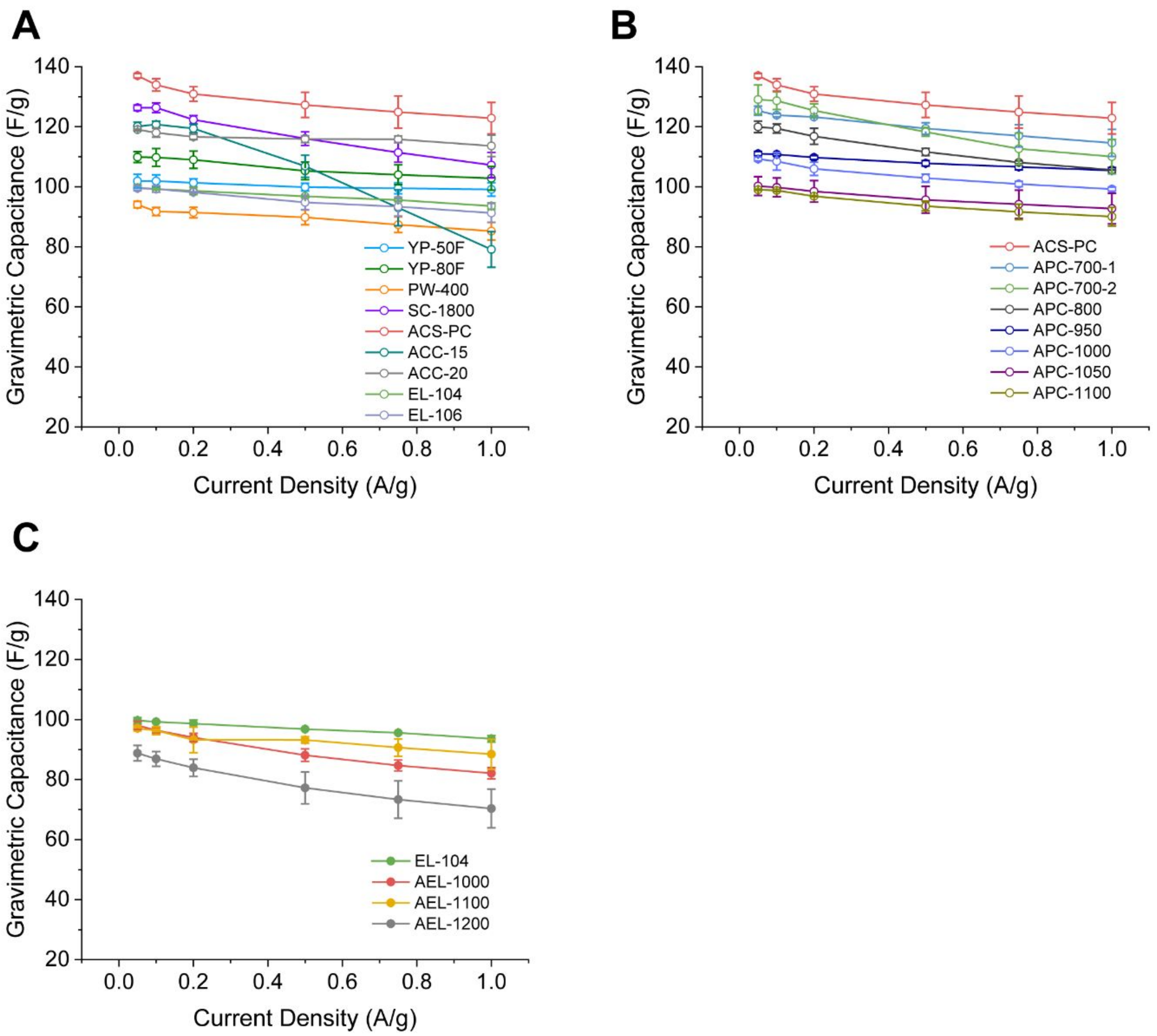

Figure S3: Gravimetric capacitance of the studied carbons at varying current densities in $EMIBF_4$. (**A**) Commercial activated carbons. **(B)** Pristine ACS-PC and thermally annealed ACS-PC at varying temperatures. **(C)** Pristine EL-104 and thermally annealed EL-104 at varying temperatures.

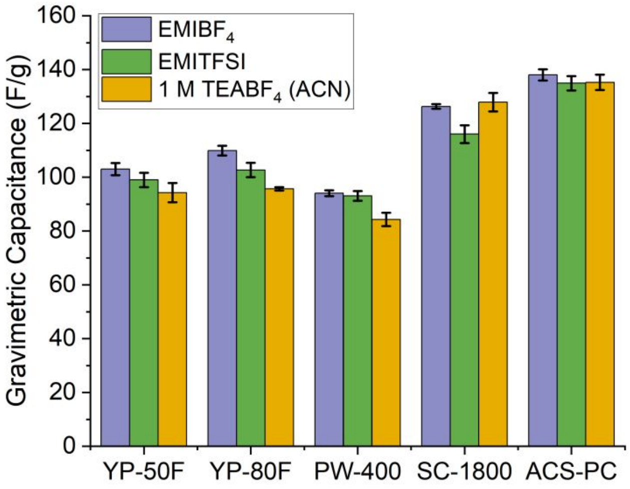

Figure S4: Gravimetric capacitance of five commercial activated carbons in different electrolytes.

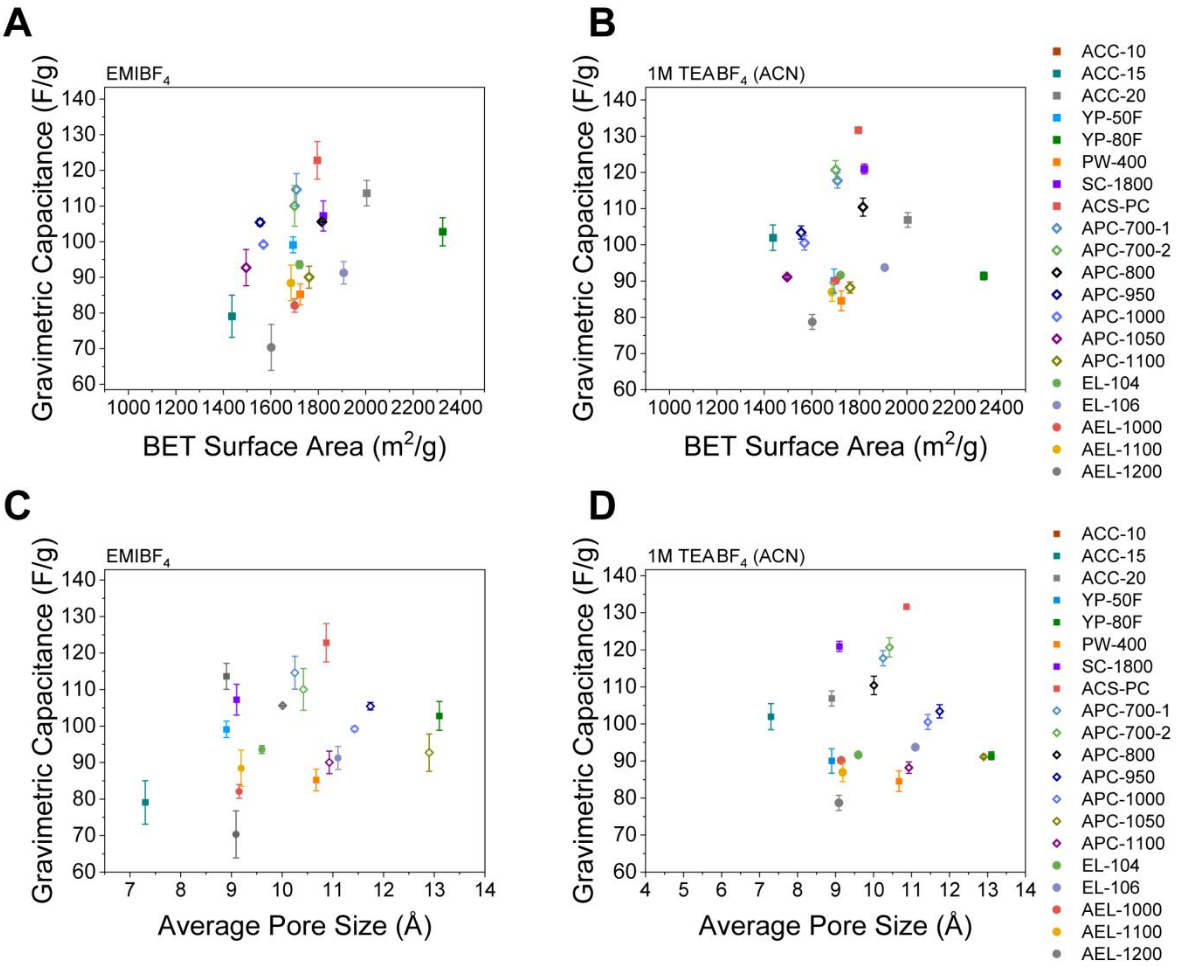

Figure S5: Relationship between gravimetric capacitances at 1 A/g and pore structures of the studied carbons. (**A**) Relationship between gravimetric capacitance at 1 A/g in $EMIBF_4$ and BET surface area. **(B)** Relationship between gravimetric capacitance at 1 A/g in 1 M $TEABF_4$ and BET surface area. (**C**) Relationship between gravimetric capacitance at 1 A/g in $EMIBF_4$ and average pore size. (**D**) Relationship between gravimetric capacitance at 1 A/g in $EMIBF_4$ and average pore size. The results of BET surface area, average pore size and capacitance values in 1 M TEABF4 (ACN) were adapted from our previous work.[1]

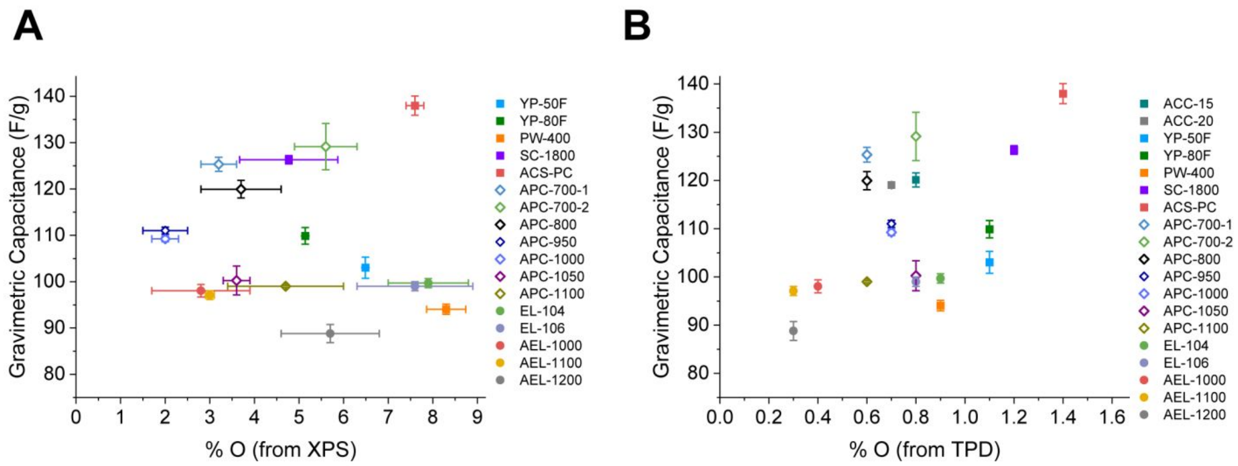

Figure S6: (A) Relationship between gravimetric capacitances at 0.05 A/g and oxygen contents measured with XPS experiments, with error bars representing the standard deviation between the spectra acquired from 2-3 different spots on each sample. **(B)** Relationship between gravimetric capacitances at 0.05 A/g and oxygen contents measured with TPD experiments.

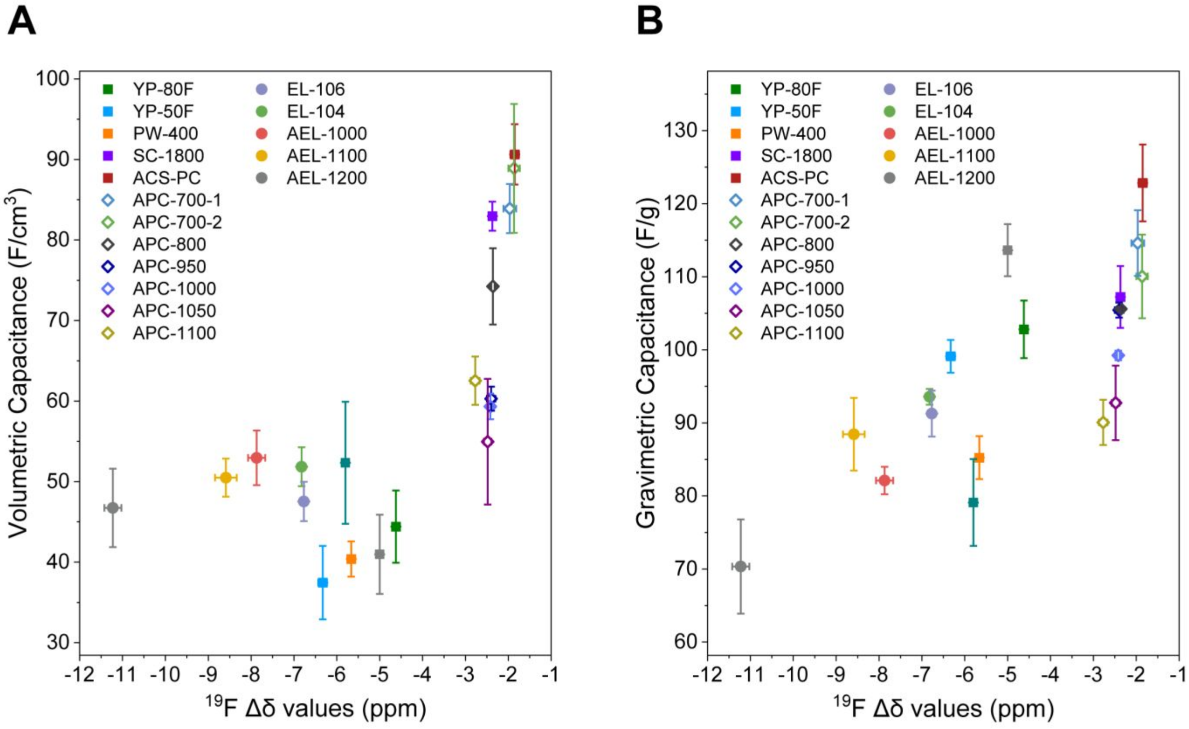

Figure S7: (**A**) Relationship between volumetric capacitance at 0.05 A/g in $EMIBF_4$ and ^{19}F $\Delta\delta$ values for all the studied carbons. **(B)** Relationship between gravimetric capacitance at 1 A/g in $EMIBF_4$ and ^{19}F $\Delta\delta$ values for all the studied carbons.

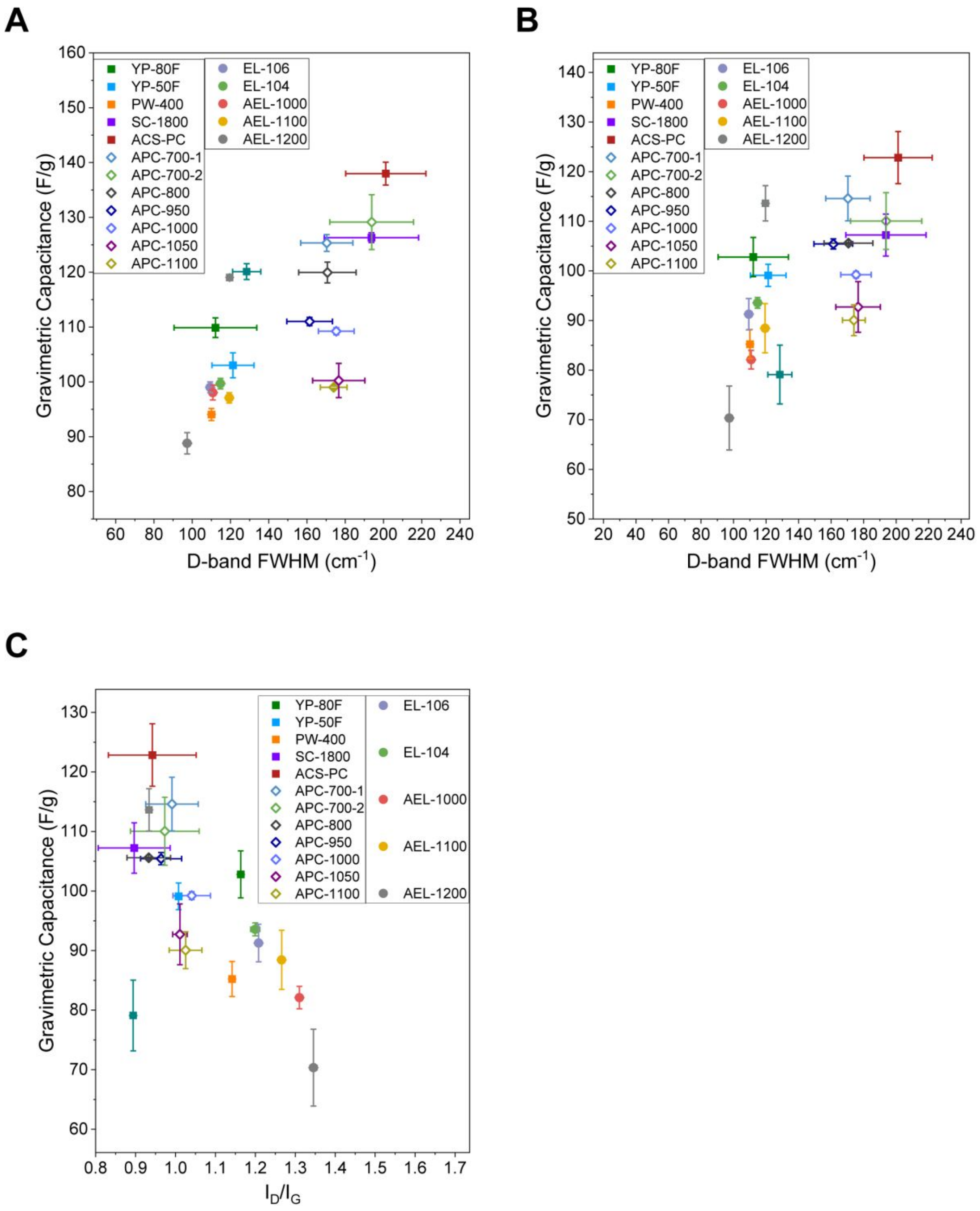

Figure S8: Relationship between gravimetric capacitances in $EMIBF_4$ and Raman spectroscopy parameters (**A**) Relationship between gravimetric capacitance in $EMIBF_4$ at 0.05 A/g and D-band full width half maximum for all studied nanoporous carbons. **(B)** Relationship between gravimetric capacitance in $EMIBF_4$ at 1 A/g and D-band full width half maximum for all studied nanoporous carbons. **(C)** Relationship between gravimetric capacitance in $EMIBF_4$ at 1 A/g and I_D/I_G values for all studied nanoporous carbons. The D-band full width half maximum and I_D/I_G values of all the studied carbons were adapted from our previous work.[4]

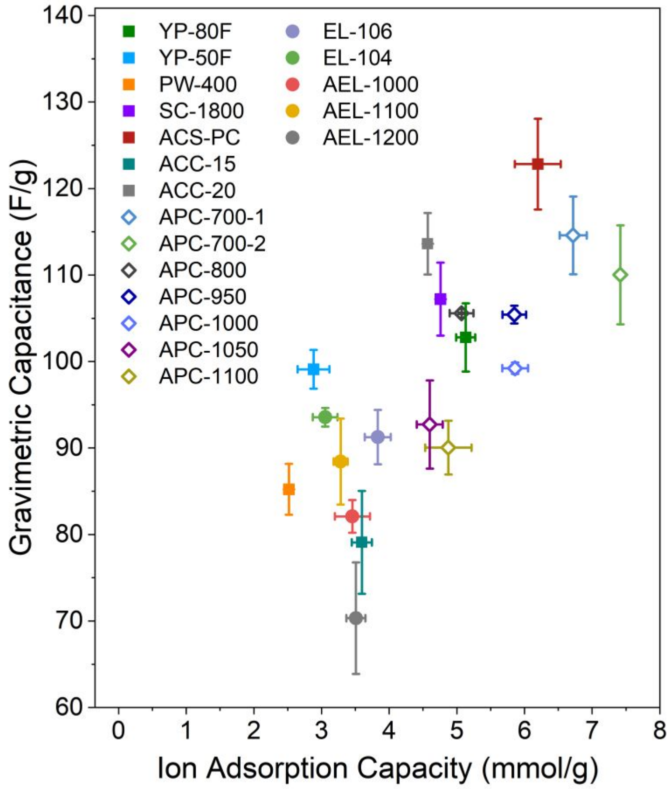

Figure S9: Relationship between gravimetric capacitances at 1 A/g in $EMIBF_4$ and ion adsorption capacities (as measured by ^{19}F NMR) for all the studied carbons.

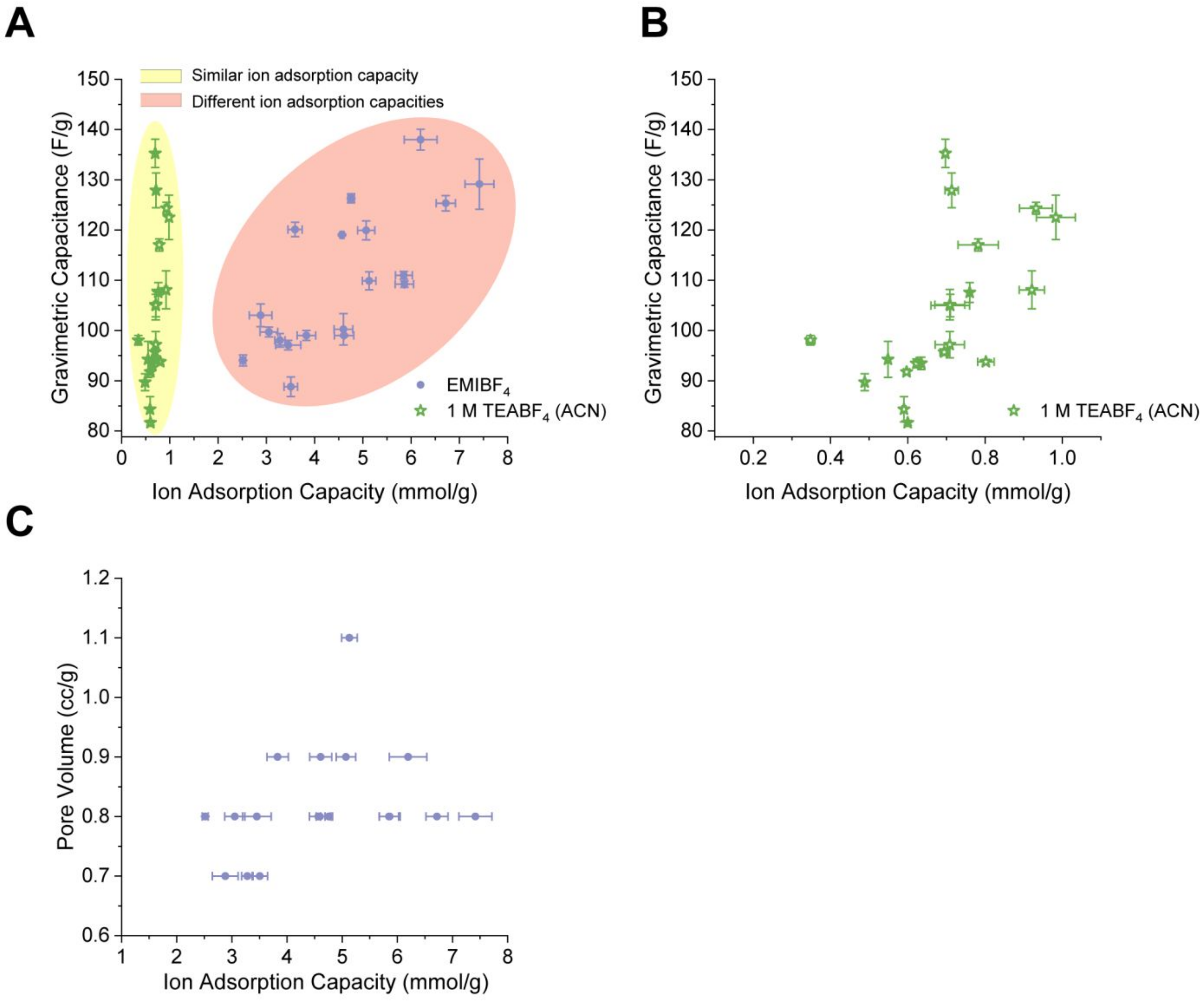

Figure S10: Relationship between gravimetric capacitances at 0.05 A/g and ion adsorption capacities (as measured by ^{19}F NMR) in $EMIBF_4$ and 1 M $TEABF_4$ (ACN) for all the studied carbons. (**B**)Relationship between gravimetric capacitances at 0.05 A/g and ion adsorption capacities (as measured by ^{19}F NMR) in 1 M $TEABF_4$ (ACN). The data in 1 M $TEABF_4$ (ACN) were adapted from our previous work.[1] The points highlighted in yellow show nanoporous carbons have similar ion adsorption capacities in 1 M $TEABF_4$ (ACN), in which case the capacitance is dominated by the graphene-like domain sizes as show in Figure 2d and our previous study.[1] (**C**) Relationship between pore volume derived from N_2 gas physisorption measurements and ion adsorption capacity (as measured by ^{19}F NMR) in $EMIBF_4$.

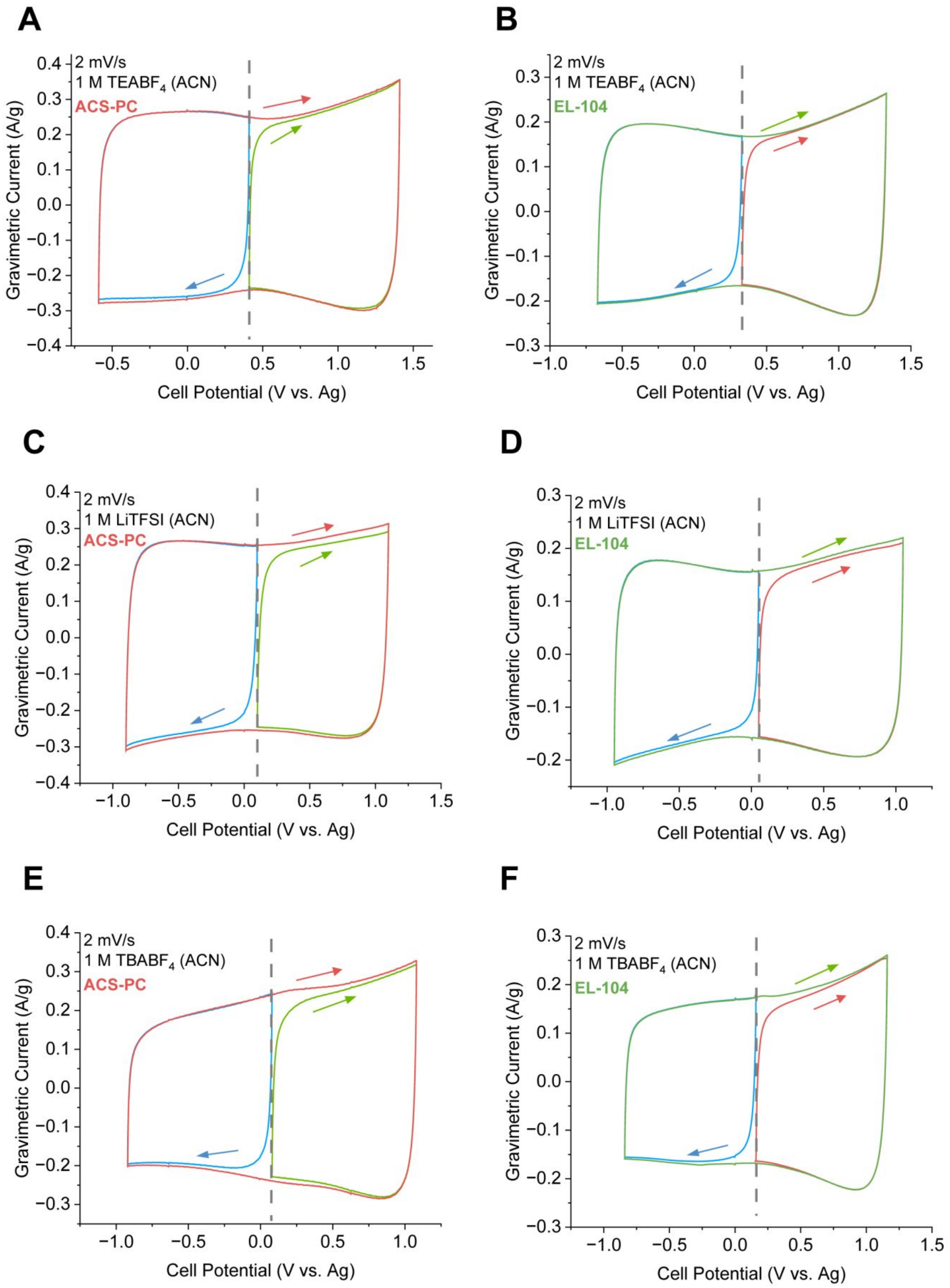

Figure S11: Cyclic voltammograms obtained at 2 mV/s from three-electrode cell with ACS-PC as working electrode, an oversized YP-80F counter electrode, and an Ag pseudo-reference electrode in **(A)** 1 M $TEABF_4$ (ACN). **(C)** 1 M LiTFSI (ACN) and **(E)** 1 M $TBABF_4$ (ACN). Cyclic voltammograms obtained at 2 mV/s from three-electrode cell with EL-104 as working

electrode, YP-80F as counter electrode and Ag pseudo-reference electrode in **(B)** 1 M $TEABF_4$ (ACN). **(D)** 1 M LiTFSI (ACN) and **(F)** 1 M $TBABF_4$ (ACN). The open circuit voltage is indicated by the grey dashed line. CV was obtained by scanning to +1 V vs. E_{OCV} to -1 V vs. E_{OCV} and across the full potential window. The direction of scanning is indicated by the arrow in each case. The potential of zero charge read from CV is 0.57 and 0.10 V vs. Ag in 1 M $TEABF_4$ (ACN) and 1 M LiTFSI (ACN) for ACS-PC and 0.40 and -0.01 V vs. Ag in 1 M $TEABF_4$ (ACN) and 1 M LiTFSI (ACN) for EL-104. Despite different carbons having different PZCs in different electrolytes, the capacitance for a given carbon remains similar, provided that the electrolyte ions can access the pores.

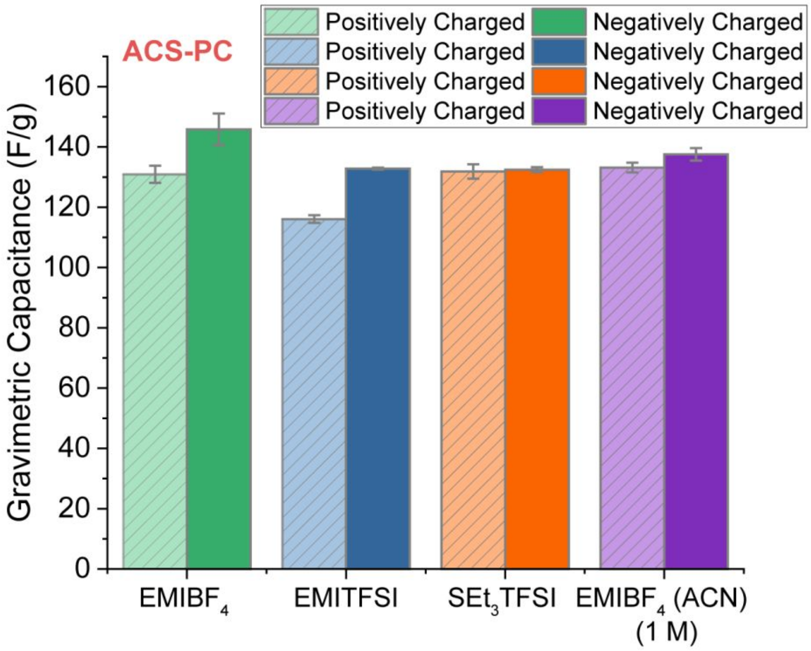

Figure S12: Gravimetric capacitances of ACS-PC calculated from constant charge-discharge measurements at 0.05 A/g in a three-electrode cell in $EMIBF_4$, EMITFSI, SEt_3TFSI and 1 M $EMIBF_4$ (ACN).

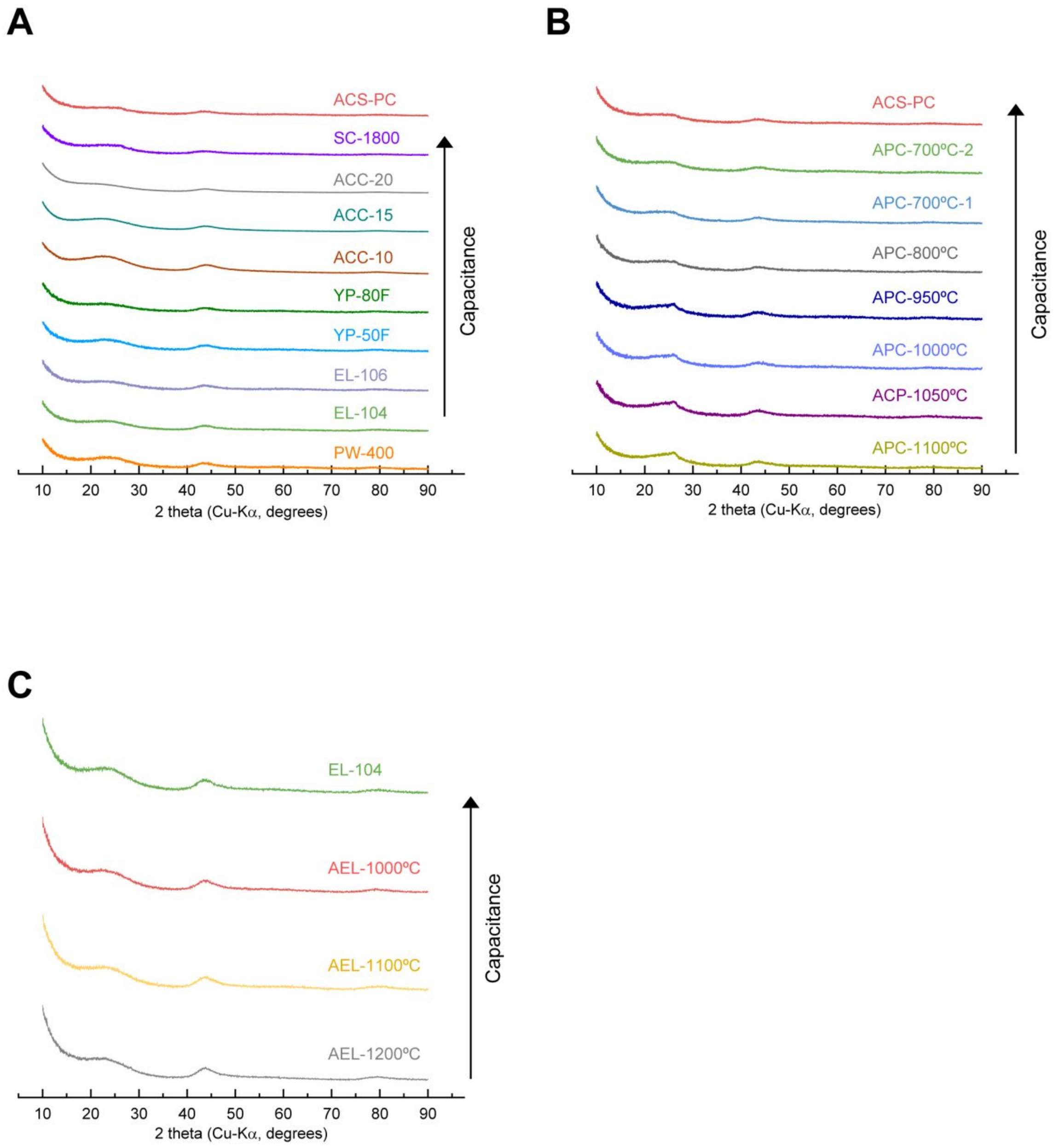

Figure S13: X-ray diffraction patterns for nanoporous carbons studied in this work. (**A**) Commercial activated nanoporous carbons. (**B**) ACS-PC and thermally annealed ACS-PC at different temperatures. (**C**) EL-104 and thermally annealed EL-104 at different temperatures.

Supplementary Tables

Table S1: BET surface area, accumulative pore volume and average pore size of all the studied carbons, derived from N_2 isotherms (77 K), adapted from our previous work.

Carbon Name	BET Surface Area (m^2/g)	Accumulative Pore Volume (cc/g)	Average Pore Size (Å)
YP-50F	1694	0.7	8.9
YP-80F	2264	1.1	13.1
PW-400	1724	0.8	10.7
SC-1800	1821	0.8	9.1
ACS-PC	1796	0.9	10.9
EL-104	1721	0.8	9.6
EL-106	1907	0.9	11.1
ACC-10	1094	0.4	5.6
ACC-15	1436	0.5	7.3
ACC-20	2004	0.8	8.9
APC-700-1	1708	0.8	10.3
APC-700-2	1700	0.8	10.4
APC-800	1815	0.9	10.0
APC-950	1555	0.8	11.7
APC-1000	1569	0.8	11.4
APC-1050	1496	0.8	12.9
APC-1100	1761	0.9	10.9
AEL-1000	1701	0.8	9.2
AEL-1100	1685	0.7	9.2
AEL-1200	1602	0.7	9.1

Table S2: Chemical shift of in-pore resonances and ^{19}F $\Delta\delta$ value for all studied carbons in $EMIBF_4$.

Carbon Name	In-pore resonance chemical shift (ppm)	^{19}F $\Delta\delta$ value (ppm)
YP-50F	-154.83±0.10	-6.33±0.10
YP-80F	-153.16±0.03	-4.66±0.03
PW-400	-154.16±0.1	-5.66±0.1
SC-1800	-150.53±0.05	-2.03±0.05
ACS-PC	-150.39±0.15	-1.89±0.15
EL-104	-155.32±0.07	-6.82±0.07
EL-106	-155.27±0.02	-6.77±0.02
ACC-15	-154.30±0.02	-5.80±0.02
ACC-20	-153.50±0.07	-5.00±0.07
APC-700-1	-150.46±0.15	-1.96±0.15
APC-700-2	-150.36±0.13	-1.86±0.13
APC-800	-150.86±0.04	-2.36±0.04
APC-950	-150.90±0.10	-2.40±0.10
APC-1000	-150.92±0.04	-2.42±0.04
APC-1050	-150.98±0.08	-2.48±0.08
APC-1100	-151.27±0.10	-2.77±0.10
AEL-1000	-156.37±0.20	-7.87±0.20
AEL-1100	-157.09±0.25	-8.59±0.25
AEL-1200	-159.73±0.23	-11.23±0.23

Table S3: Ion size of the four electrolytes.[7, 8]

Electrolyte	Cation Size (nm)	Anion Size (nm)
1 M $TBABF_4$ (ACN)	0.82 (bare)<br>1.44 (solvated)	0.48 (bare)<br>1.16 (solvated)
1 M $TEABF_4$ (ACN)	0.68 (bare)<br>1.3 (solvated)	0.48 (bare)<br>1.16 (solvated)
$EMIBF_4$	0.76	0.48
EMITFSI	0.76	0.79

Supplementary References

(1) Liu, X. Y.; Lyu, D. X.; Merlet, C.; Leesmith, M.; Hua, X.; Xu, Z.; Grey, C. P.; Forse, A. C. Structural disorder determines capacitance in nanoporous carbons. *Science* **2024**, *384* (6693), 321-325. DOI: 10.1126/science.adn6242.
(2) Forse, A. C.; Griffin, J. M.; Merlet, C.; Bayley, P. M.; Wang, H.; Simon, P.; Grey, C. P. NMR Study of Ion Dynamics and Charge Storage in Ionic Liquid Supercapacitors. *J Am Chem Soc* **2015**, *137* (22), 7231-7242. DOI: 10.1021/jacs.5b03958.
(3) Asres, N. E.; Mysyk, R.; Gómez, K.; Carriazo, D.; López del Amo, J. M. Understanding the effect of nanosized carbon texture on ion adsorption and electrochemical response via in-situ time-evolution solid-state NMR. *Energy Storage Materials* **2025**, *77*, 104172. DOI: 10.1016/j.ensm.2025.104172.
(4) Liu, X. Y.; Choi, J.; Xu, Z.; Grey, C. P.; Fleischmann, S.; Forse, A. C. Raman Spectroscopy Measurements Support Disorder-driven Capacitance in Nanoporous Carbons. *J Am Chem Soc* **2024**, *146* (45), 30748-30752. DOI: 10.1021/jacs.4c10214.
(5) Ishii, T.; Kashihara, S.; Hoshikawa, Y.; Ozaki, J.-i.; Kannari, N.; Takai, K.; Enoki, T.; Kyotani, T. A quantitative analysis of carbon edge sites and an estimation of graphene sheet size in high-temperature treated, non-porous carbons. *Carbon* **2014**, *80*, 135-145. DOI: 10.1016/j.carbon.2014.08.048.
(6) Yoshii, T.; Nishikawa, G.; Prasad, V. K.; Shimizu, S.; Kawaguchi, R.; Tang, R.; Chida, K.; Sato, N.; Sakamoto, R.; Takatani, K.; et al. Quantitative and qualitative analysis of nitrogen species in carbon at the ppm level. *Chem* **2024**, *10* (8), 2450-2463. DOI: 10.1016/j.chempr.2024.03.029 (acccessed 2025/02/17).
(7) Largeot, C.; Portet, C.; Chmiola, J.; Taberna, P. L.; Gogotsi, Y.; Simon, P. Relation between the ion size and pore size for an electric double-layer capacitor. *J Am Chem Soc* **2008**, *130* (9), 2730-+. DOI: 10.1021/ja7106178.
(8) Ue, M. Mobility and Ionic Association of Lithium and Quaternary Ammonium Salts in Propylene Carbonate and γ-Butyrolactone. *J Electrochem Soc* **1994**, *141* (12), 3336. DOI: 10.1149/1.2059336.
